# POSTPARTUM GAD IS A RISK FACTOR FOR POSTPARTUM MDD: THE COURSE AND LONGITUDINAL RELATIONSHIPS OF POSTPARTUM GAD AND MDD

**DOI:** 10.1002/da.22040

**Published:** 2013-01-03

**Authors:** Jason Prenoveau, Michelle Craske, Nicholas Counsell, Valerie West, Beverley Davies, Peter Cooper, Elizabeth Rapa, Alan Stein

**Affiliations:** 1 Department of Psychology, Loyola University MarylandBaltimore, Maryland; 2 Department of Psychology and Department of Psychiatry & Biobehavioral Sciences, University of CaliforniaLos Angeles, California; 3 Department of Child and Adolescent Psychiatry, University of OxfordUnited Kingdom; 4 School of Psychology and Clinical Language Sciences, the University of ReadingUK; and Department of Psychology, Stellenbosch UniversitySouth Africa

**Keywords:** anxiety disorders, depressive disorders, depression, postpartum, comorbidity, diagnosis, prospective studies, epidemiology

## Abstract

**Background:**

The objective was to examine the course and longitudinal associations of generalized anxiety disorder (GAD) and major depressive disorder (MDD) in mothers over the postpartum 2 years.

**Method:**

Using a prospective naturalistic design, 296 mothers recruited from a large community pool were assessed for GAD and MDD at 3, 6, 10, 14, and 24 months postpartum. Structured clinical interviews were used for diagnoses, and symptoms were assessed using self-report questionnaires. Logistic regression analyses were used to examine diagnostic stability and longitudinal relations, and latent variable modeling was employed to examine change in symptoms.

**Results:**

MDD without co-occurring GAD, GAD without co-occurring MDD, and co-occurring GAD and MDD, displayed significant stability during the postpartum period. Whereas MDD did not predict subsequent GAD, GAD predicted subsequent MDD (in the form of GAD + MDD). Those with GAD + MDD at 3 months postpartum were significantly less likely to be diagnosis free during the follow-up period than those in other diagnostic categories. At the symptom level, symptoms of GAD were more trait-like than those of depression.

**Conclusions:**

Postpartum GAD and MDD are relatively stable conditions, and GAD is a risk factor for MDD but not vice versa. Given the tendency of MDD and GAD to be persistent, especially when comorbid, and the increased risk for MDD in mothers with GAD, as well as the potential negative effects of cumulative exposure to maternal depression and anxiety on child development, the present findings clearly highlight the need for screening and treatment of GAD in addition to MDD during the postpartum period.

## INTRODUCTION

In contrast to the substantial research on maternal major depressive disorder (MDD) during the postpartum period,^1^ much less research has evaluated postpartum anxiety. Yet one particular anxiety disorder, generalized anxiety disorder (GAD), may be especially relevant during the postpartum period, given that family issues, including child wellbeing, are a common source of excessive worrying in persons with GAD.^2^ Although the high rate of comorbidity between MDD and GAD is well recognized,^3^ few studies have evaluated the relationship between GAD and MDD in prospective longitudinal designs, and to our knowledge none has evaluated their relationships in the postpartum period. Because childbirth is a common major life-stressor, the 2 years following childbirth is a good opportunity to evaluate and compare the course of these disorders.

The temporal course of GAD and MDD is relevant to the ongoing debate about whether these two disorders represent different subtypes of the same disorder as opposed to being truly distinct disorders.^4^ The postpartum period provides a major life stress interval over which the chronological course of psychiatric disorders can be examined. Evidence regarding the temporal relationship between GAD and MDD will contribute to ongoing discussion about the diagnostic boundaries between these two disorders.^5^ On one hand, there are a number of shared elements across GAD and MDD, which argues for them being subtypes of the same disorder. Shared elements of these highly comorbid conditions include familial transmission, genetic risk, neuroticism, and childhood risk factors such as abuse and parental divorce.^3^ In addition, they share demographic features such as higher rates in females, middle age, and low socioeconomic class,^3^ and similar ages of onset.^6^ On the other hand, familial aggregation is stronger for MDD than GAD,^3^,^7^ childhood abuse is more prevalent in GAD than MDD,^7^ and there is a stronger association between GAD and bipolar disorders than between GAD and MDD.^8^ Furthermore, neuroimaging and neuroendocrine studies discriminate the two disorders.^9^ Finally, temporal asymmetry has been reported, where GAD and MDD are comorbid, GAD is more likely to have preceded MDD than vice versa,^10^–^12^ although there are exceptions.^13^ However, the majority of the research on this important issue of temporal sequencing derives from retrospective reporting, with few prospective longitudinal investigations^12^,^13^ and none within the postpartum period. The current study aims to address this limitation by evaluating the temporal sequence between maternal GAD and MDD in a prospective longitudinal design, from the reference point of childbirth.

Another reason to study postpartum MDD and GAD is because of the possible implications for child development. There is a great deal of evidence that postnatal depression is associated with negative child outcomes^1^ and that the longer the period of maternal depression in the first few years of life, the more negative the effect on the child's development, including cognitive and social development, behavior, and attachment.^14^–^19^ However, these studies have rarely made systematic assessment of maternal anxiety. Given the strong association between depression and anxiety,^6^ it is possible that some of the observed effects on child outcome are due, in part, to maternal anxiety or to the shared features of depression and anxiety. Also, there is emerging evidence that postpartum maternal GAD is associated with adverse childhood outcomes independent of maternal depression.^20^ Additionally, since the co-occurrence of GAD and MDD is associated with greater functional impairment than either disorder alone,^21^ it is possible that co-occurring GAD and MDD confers particular risk for negative outcomes among offspring. Thus, it is critical to understand the course of GAD, MDD, and the interrelationship of the two disorders to inform strategies for screening and treatments in postpartum mothers.

In samples *not* selected specifically from a maternal postpartum population, prospective longitudinal studies have demonstrated significant stability of MDD and GAD, with some showing greater stability of co-occurring MDD and GAD relative to pure states.^12^ In samples specific to postpartum mothers, studies have demonstrated that depression is relatively stable at both symptom^22^,^23^ and syndrome^18^,^24^,^25^ levels. However, as most studies have not examined maternal anxiety, it is possible that stability of co-occurring depression and anxiety contributes to some of the observed stability in depression.

The aims of the current study were to examine the course and interrelationship of MDD and GAD from 3 (3M-P) to 24 months postpartum (24M-P). At the syndrome level, stability and longitudinal relations were examined for diagnoses of GAD without co-occurring MDD (GAD-only), MDD without co-occurring GAD (MDD-only), and co-occurring GAD and MDD. Stability and longitudinal relations were also examined at the symptom level using a latent variable measurement model because such modeling provides constructs that are theoretically free of measurement error and allows for testing of longitudinal measurement invariance (i.e., enables testing of whether or not study measures are functioning the same at different time points). Furthermore, trait–state–occasion (TSO)^26^ modeling was used for symptom constructs to determine how much of their variability was stable across all time points (represented by a trait component), how much was predicted from the prior time point (represented by an autoregressive occasion pathway), and how much was residual or unexplained.

## METHOD

### PARTICIPANTS

Participants were mothers recruited from postnatal wards at the John Radcliffe Hospital, Oxford as well as a number of health centers in Oxfordshire as part of the larger Oxford Parent Project (OPP). Mothers were eligible if they were 18 years or older, spoke sufficient English, lived within 35 miles of Oxford, had no life-threatening medical conditions, planned to be the infant's principal caretaker, and had delivered an infant over 35 weeks gestation, over 2,000 grams birth weight, and with no life-threatening medical complications. Study approval was obtained from the Oxford Research Ethics Committee; after complete description of the study, written informed consent was obtained from all potential participants.

When their infants were approximately 9 weeks old, 2,202 mothers completed screening questionnaires (the Edinburgh Postnatal Depression Scale (EPDS) and Generalized Anxiety Disorder Questionnaire (GAD-Q), described below) in order to identify those who likely had MDD, GAD, or both. To ensure there were adequate numbers of participants with, and without, these disorders for the OPP, mothers who scored high on either screening questionnaire, and a randomly selected group of women who scored below the cutoff on both questionnaires, were selected for diagnostic interviews (Structured Clinical Interview for DSM diagnosis (SCID); see below) at 3M-P; see^20^ for further details on participant selection. Demographic information for the 296 participants who completed an assessment at 3M-P is presented in Table 1; demographic information is presented separately by diagnostic status at 3M-P (diagnostic assessment details presented in the Measures and Procedures sections below).

**TABLE 1:** Demographics by diagnostic status at 3 months postpartum

Demographic variable	Variable level	Co-occurring GAD and MDD (*n* = 41)	GAD-only (*n* = 80)	MDD-only (*n* = 40)	No diagnosis (*n* = 135)	Statistics
Mother age in years, mean (*SD*)		32.5 (5.3)	31.8 (5.1)	32.3 (5.6)	32.6 (4.8)	*F*(3,292) = 0.4, *P* = .74
Infant age in months, mean (*SD*)		3.5 (.9)	3.7 (.8)	3.7 (1.2)	3.5 (.6)	*F*(3,319) = 1.3, *P* = .26
Infant sex, frequency (%)	Female	17 (41.5%)	40 (50%)	21 (52.5%)	70 (51.9%)	
	Male	24 (58.5%)	40 (50%)	19 (47.5%)	65 (48.1%)	χ^2^(3) = 1.5, *P* = .69
Infant birth order, frequency (%)	First born	19 (46.3%)	48 (60.0%)	19 (47.5%)	94 (69.6%)	
	Not first born	22 (53.7%)	32 (40.0%)	21 (52.5%)	41 (30.4%)	χ^2^(3) = 11.0, *P* = .01
Mother marital status, frequency (%)	Married	19 (46.3%)	48 (60.0%)	24 (60.0%)	93 (68.9%)	
	Other	5 (12.2%)	22 (27.5%)	9 (22.5%)	25 (18.5%)	
	Missing	17 (41.5%)	10 (12.5%)	7 (17.5%)	17 (12.6%)	χ^2^(3) = 23.0, *P* = .001

### MEASURES

The EPDS was administered and is a well-validated 10-item questionnaire that assesses depressive symptoms in early motherhood.^27^ The GAD-Q was also administered and is a well-validated 9-item questionnaire that assesses symptoms of GAD.^28^ The SCID is a semistructured interview for diagnosing psychiatric disorders that has demonstrated satisfactory reliability and validity.^29^ Clinician Severity Ratings (CSRs) provide a measure of the extent of symptom severity, distress, and impairment associated with psychiatric disorders, using a 0–8 points scale (with scores of 4 or greater indicating clinical severity). CSRs have demonstrated good interrater reliability.^30^ Interviewers underwent extensive SCID and CSR training; supervision and review of diagnostic assessments was led by one principal investigator (A.S.) in weekly case meetings, and another principal investigator (M.G.C.) reviewed tapes of randomly selected interviews. Following initial training, after each interviewer completed four to five interviews, independent interviewers co-rated the next interview to assure interrater reliability.

### PROCEDURE

The EPDS and GAD-Q were used to screen for symptoms of depression and GAD at 9 weeks. 3M-P assessments were conducted at participants’ homes when their infants were 3 months old and consisted of administering the SCID (to assess diagnostic status and CSRs). At all subsequent assessments, mothers were reinterviewed using the SCID and completed EPDS and GAD-Q; these assessments occurred at approximately 6 months postpartum (6M-P), 10 months postpartum (10M-P), 14 months postpartum (14M-P), and 24M-P. Mothers assessed as not having MDD or GAD at 3M-P were only included in the study (as the no-diagnosis group) if they also did not have a history of psychiatric illness. The following number of participants provided data: 296 at 6M-P, 265 at 10M-P, 233 at 14M-P, and 234 at 24M-P.

## RESULTS

### DIAGNOSTIC STABILITY AND ASSOCIATIONS

Diagnostic stability was examined by grouping individuals at each time point into one of four mutually exclusive categories based on SCID diagnosis: those who did not meet DSM diagnostic criteria for either MDD or GAD (no diagnosis), those who met DSM-IV diagnostic criteria for MDD only (MDD-only), for GAD only (GAD-only), and for both GAD and MDD (GAD + MDD). Those in a given diagnostic category at 3M-P were generally significantly more likely to fall into that same category at least once during the follow-up period than those who were not in that category at 3M-P (see Table 2); the exception was that GAD-only at 3M-P was not significantly more likely to have GAD-only during follow-up than GAD + MDD at 3M-P. Further, those in a given diagnostic category at 3M-P generally fell into that same category on a greater number of follow-up time points than those who were not in that category at 3M-P (see Table 2); again, the only exception was that 3M-P GAD-only did not differ from 3M-P GAD + MDD in number of follow-up time points diagnosed with GAD-only.

**TABLE 2:** Diagnostic status at follow-up as a function of diagnostic status at 3 months postpartum

	Co-occurring GAD and MDD		GAD-only		MDD-only		No diagnosis		Missing data	
					
Diagnostic status at 3 months postpartum	*N* (%) with dx at least 1 follow-up time point	*M* (*SD*) of no. of follow-up time points with dx	*N* (%) with dx at least 1 follow-up time point	*M* (*SD*) of no. of follow-up time points with dx	*N* (%) with dx at least 1 follow-up time point	*M* (*SD*) of no. of follow-up time points with dx	*N* (%) with no dx at least 1 follow-up time point	*M* (*SD*) of no. of follow-up time points with no dx	*N* (%) missing data at least 1 follow-up time point	*M* (*SD*) of no. of follow-up time points with missing data
GAD and MDD	26	1.3	21	0.8_e_	5	0.1_h_	19	1.1_j_	14_k_	0.6_l_
	(63.4%)	(1.3)	(51.2%)_d_	(1.0)	(12.2%)_g_	(0.4)	(46.3%)	(1.4)	34.1%	(1.0)
GAD-Only	18	0.4_c_	47	1.1_e_	4	0.1_h_	59	1.8_j_	27_k_	0.6_l_
	(22.5%)_a_	(0.8)	(58.8%)_d_	(1.2)	(5.0%)_g_	(0.3)	(73.8%)_i_	(1.5)	33.8%	(1.0)
MDD-Only	5	0.1_c_	7	0.3_f_	21	1.0	34	1.8_j_	17_k_	0.8_l_
	(12.5%)_a,b_	(0.3)	(17.5%)	(0.6)	(52.5%)	(1.2)	(85.0%)_i_	(1.3)	42.5%	(1.1)
No diagnosis	5	0.1_c_	4	0.1_f_	5	0.1_h_	133	3.4	29_k_	0.4_l_
	(3.7%)_b_	(0.4)	(3.0%)	(0.3)	(3.7%)_g_	(0.5)	(98.5%)	(1.0)	21.5%	(0.8)

*Note:* Follow-up time points include 6, 10, 14, and 24 months postpartum. Shared alphabetical subscripts represent values that are not significantly different from each other within columns. For columns with frequency data, 4 (T1 diagnostic category) × 2 (presence versus absence of the dx category under consideration for at least one follow-up time point) contingency tables were examined for each diagnostic category at follow-up. Chi-square tests were significant for all follow-up diagnostic categories, all χ^2^(3) > 66.3, *P* < .001, as well as for missing data, χ^2^(3) = 8.6, *P* = .04. Pairwise comparisons were then conducted within follow-up diagnostic categories using two-sided Fisher's exact tests with a Bonferroni corrected α of 0.0083 (six pairwise comparisons per column); alphabetical subscripts above provide the results of these comparisons. For columns with mean number of follow-up time points, Kruskal–Wallis tests were employed due to data nonnormality. Omnibus tests were significant at each follow-up diagnostic category, all χ^2^(3) > 77.6, *P* < .001, as well as for missing data, χ^2^(3) = 8.0, *P* = .05. Results from pairwise comparisons^32^ conducted within follow-up diagnostic categories (with alpha corrected for six pairwise comparisons) are presented with alphabetical subscripts above.

**Figure 1:**
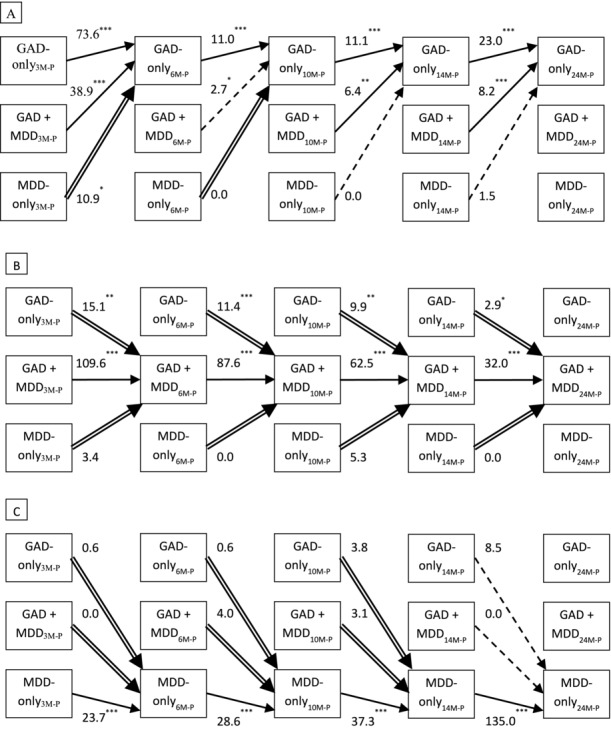
Longitudinal associations (odds ratios) among diagnostic categories based on pairwise logistic regression of adjacent time points. Odds ratios are the odds of those with the diagnosis under consideration at the preceding time having the predicted diagnosis at the subsequent time point divided by the odds of those with no diagnosis at the preceding time having the predicted diagnosis at the subsequent time. GAD-only, Generalized Anxiety Disorder (GAD) without co-occurring Major Depressive Disorder (MDD); MDD-only, MDD without co-occurring GAD (A); GAD + MDD (B), co-occurring GAD and MDD (C); 3M-P, 3 months postpartum; 6M-P, 6 months postpartum; 10M-P, 10 months postpartum; 14M-P, 14 months postpartum; 24M-P, 24 months postpartum; *P < .05; **P < .01; and **P < .001. For each adjacent time-point pairing, dashed lines represent pathways that are significantly different from the indicator pathway (e.g., for Figure 1A, GAD-Only→GAD-Only) at *P* < .05. For each adjacent time-point pairing, compound lines represent pathways that are significantly different from the indicator pathway at *P* < .01.

Logistic regression analyses using maximum likelihood estimation were conducted for adjacent time-point pairings using Mplus version 5.0 statistical software^31^ and are presented in Fig. 1; odds ratios calculated from the regression coefficients represent the odds for the given category divided by the odds of the no diagnosis category.^1^ For example, the odds ratio associated with the path from 3M-P GAD-only to 6M-P GAD-only is 73.6 meaning the odds for GAD-only at 6M-P are 73.6 times greater for the 3M-P GAD-only group than for the 3M-P no diagnosis group.

As seen in Fig. 1A, both GAD-only and GAD + MDD were significantly associated with the presence of GAD-only at each subsequent time point whereas MDD-only was generally not associated with future GAD-only. Wald tests of equality constraints were examined to determine if the strength of association with subsequent GAD-only was significantly different for different diagnostic categories. The magnitude of GAD-only→GAD-only associations was always larger than GAD + MDD→GAD-only associations. However, as seen in Fig. 1A (dashed line), constraining these parameters to be equal revealed that the difference between the two only rose to significance for the 6M-P to 10M-P pairing, Δχ^2^(1) = 4.3, *P* = .04. As seen in Fig. 1A (dashed and compound lines), the magnitude of GAD-only→GAD-only associations were significantly greater than MDD-only→GAD-only for all time-point pairings, all Δχ^2^(1) > 5.6, *P* < .02.

As seen in Fig. 1B, both GAD-only and GAD + MDD were significantly associated with GAD + MDD at subsequent time points whereas MDD-only never was. Also seen in Fig. 1B (compound lines), Wald tests of equality constraints revealed that GAD + MDD→GAD + MDD associations were significantly larger than those of GAD-only→GAD + MDD for all time-point pairings, all Δχ^2^(1) > 7.2, *P* < .01, and MDD-only→GAD + MDD for all time-point pairings, all Δχ^2^(1) > 7.6, *P* < .01. Fig. 1C shows that MDD-only was significantly associated with MDD-only at all subsequent time points, but that GAD-only and GAD + MDD were never significantly associated with MDD-only. Further, as seen in Fig. 1C (dashed and compound lines), the strength of association between MDD-only→MDD-only was significantly greater than that of GAD-only→MDD-only for all time-point pairings, all Δχ^2^(1) > 5.3, *P* < .05, and GAD + MDD→MDD-only for all time-point pairings, all Δχ^2^(1) > 6.2, *P* < .05.

### SYMPTOM STABILITY AND ASSOCIATIONS

The latent construct representing GAD symptoms was indicated by GAD CSRs and two subscales that were created by dividing the nine GAD-Q items and two EPDS items into five- and six-item subscales.^2^ These subscales had 3M-P α reliability estimates of 0.85 and 0.88. The latent construct representing MDD symptoms was indicated by MDD CSRs, and two subscales that were created by dividing six EPDS items into two three-item subscales.^2^ These subscales had 3M-P alpha reliability estimates of 0.80 and 0.82.

Because no significant effects were found for predicting participant attrition at 6M-P, 10M-P, 14M-P, or 24M-P from 3M-P GAD or MDD symptoms, data were considered missing at random^33^ and were accommodated using full information maximum-likelihood estimation. Longitudinal measurement models were examined to ensure that the manifest variables specified were significant indicators of their latent constructs at each time point and that they were consistent indicators of these constructs with time. Such consistency is needed to ensure that change in latent constructs with time was not confounded by change in construct measurement with time. To conclude there is a good fit between the observed data and the hypothesized model, root mean square error of approximation (RMSEA)^34^ should be less than 0.06 and the comparative fit index (CFI)^35^ should be greater than 0.95. Longitudinal measurement models were a good fit to the data for both symptoms of depression, CFI = 0.99, RMSEA = 0.045, and GAD, CFI = 0.99, RMSEA = 0.038; factor loadings for all manifest variables were statistically significant. Although neither model displayed full metric invariance, both Δχ^2^(8) = 67.9, *P* < .001, both displayed partial metric invariance. Thus, most variables were consistent indicators of their respective constructs over time2.^3^

**Figure 2:**
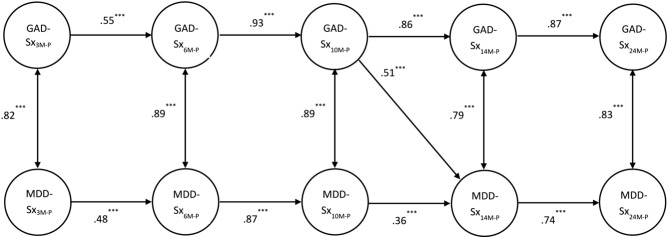
Cross-sectional and longitudinal associations (standardized path coefficients) among symptoms of Generalized Anxiety Disorder (GAD-Sx) and Major Depressive Disorder (MDD-Sx). With the exception of GAD-Sx_10M-P_ →MDD-Sx_14M-P_, all longitudinal Gad-Sx→MDD-Sx and MDD-Sx→GAD-Sx pathways have been removed because they were not significant, and removing them did not result in a significant decrement in model fit. Circles represent latent constructs from longitudinal measurement models; manifest variable indicators not shown for clarity of presentation. 3M-P, 3 months postpartum; 6M-P, 6 months postpartum; 10M-P, 10 months postpartum; 14M-P, 14 months postpartum; 24M-P, 24 months postpartum; ***P < .001.

Latent variables from longitudinal measurement models were used to examine cross-sectional and longitudinal associations among symptoms of GAD (GAD-sx) and MDD (MDD-sx). Regression pathways were included from GAD-sx and MDD-sx at each time point to both GAD-sx and MDD-sx at the subsequent time point. GAD-sx and MDD-sx were also allowed to correlate with one another at each time point so their relation at the earlier time was accounted for when predicting subsequent time points. The longitudinal GAD-sx→GAD-sx and MDD-sx→MDD-sx relations were all significant and generally of large magnitude (see Fig. 2). Although the magnitude of GAD-sx stability was greater than that of MDD-sx stability for all time points, the difference only reached significance for the last two time-point pairings, 10M-P to 14M-P, Δχ^2^(1) = 20.3, *P* < .001, and 14M-P to 24M-P, Δχ^2^(1) = 17.9, *P* < .001. After accounting for the concurrent association among GAD-sx and MDD-sx, MDD-sx did not significantly predict GAD-sx, and GAD-sx only significantly predicted MDD-sx from 10M-P to 14M-P. Further, with the exception of this significant pathway, Wald tests revealed that removal of each longitudinal cross-symptom pathway did not result in a significant decrement in model fit, all Δχ^2^(1) < 1.8, *P* > .17. Thus, these pathways were removed from the longitudinal models and from Fig. 2.

Latent variables from the longitudinal measurement models were used to examine the fit of TSO models for symptoms of GAD and MDD. Fit indices revealed that TSO models fit the data well for both MDD-sx, CFI = 0.98, RMSEA = 0.043, and GAD-sx, CFI = 0.99, RMSEA = 0.036. Pathways were added between GAD-sx and MDD-sx TSO models to examine the cross-sectional and longitudinal associations between components of the GAD-sx and MDD-sx TSO models. Unfortunately, model convergence was never achieved once pathways were included between GAD and MDD symptoms.

Wald tests were used to compare the percentages of state variance explained by model components. When comparing between constructs, the percentage of state variance explained by the trait factor was significantly greater for GAD-sx (76.5%) than MDD-sx, (54.1%), Δχ^2^(1) = 13.6, *P* < .001, and GAD-sx had significantly less unexplained state variance (21.5%) than MDD-sx (36.4%), Δχ^2^(1) = 16.7, *P* < .001.

## DISCUSSION

This study followed a community sample of mothers diagnosed in the postpartum period until their children were 24 months of age. Childbirth provides a marking life event that allows a detailed study of the course and interrelationships of MDD and GAD. This is especially important because of the possible implications for child development. At the diagnostic level, longitudinal associations indicated that GAD and MDD are distinct: MDD-only predicted MDD-only at every subsequent time point, but did not generally predict GAD-only; whereas GAD-only predicted subsequent GAD-only, but never predicted MDD-only. Such findings of longitudinal specificity in the 2 years postpartum are generally consistent with those from community samples not specific to postpartum mothers.^11^,^12^

GAD showed a significant degree of stability in mothers during the first 2 years of the postpartum period at both syndromal and symptom levels. Nearly 60% of mothers with GAD-only at 3M-P were diagnosed with GAD-only at one or more of the subsequent time points, and a diagnosis of GAD at any time point was a large predictor of subsequent GAD. The combination of GAD and MDD also demonstrated significant stability with the magnitudes of stability generally being larger than those for MDD-only and GAD-only. Further, those with GAD + MDD at 3M-P were significantly less likely to be diagnosis-free during follow-up than those with MDD-only, or GAD-only at 3M-P. The tendency for GAD + MDD to be more persistent than GAD-only or MDD-only is consistent with findings from samples not specific to postpartum mothers.^12^,^36^ Given recent evidence that postpartum maternal anxiety is associated with adverse childhood outcomes,^37^ and the possibility that GAD + MDD could confer even greater risk for adverse outcomes, the present stability findings demonstrate the importance of assessing and treating anxiety during the postpartum period in addition to depression.

Significant stability was also demonstrated for depression at both diagnostic and symptom levels. For example, over 50% of mothers with MDD-only at 3M-P were diagnosed with MDD-only at one or more of the subsequent time points. A diagnosis of MDD at any of the time points was a large predictor of subsequent MDD. Because longer periods of maternal depression during the first years of a child's life are associated with poorer child cognitive, social, and behavioral outcomes,^14^–^19^ the significant stability of maternal depression during this period further highlights the necessity of identifying and treating postpartum depression.

Although “pure” MDD and GAD generally did not predict one another, those with GAD-only were at increased risk of developing GAD + MDD, and those with GAD + MDD were likely to transition to GAD-only as their MDD remitted. However, those with MDD-only were not at increased risk of developing GAD + MDD, and those with GAD + MDD were not likely to have their GAD remit. Thus, during the 2-year postpartum period, MDD tended to be more episodic than GAD. These findings were consistent with those at the symptom level where GAD-symptoms were significantly more stable than depressive symptoms, and consistent with past research at both the syndrome^38^ and symptom^39^ levels. In addition to speaking to the relative stability of these constructs, and therefore evidence of differentiation, such findings also speak to the temporal sequencing of GAD and MDD. In postpartum mothers, GAD-only tends to precede MDD (in the form of GAD + MDD), but MDD-only does not tend to precede GAD (either GAD-only or GAD + MDD). Thus, GAD may potentially serve as a risk factor for the subsequent development of MDD, which has been found in other studies conducted outside the postpartum period.^10^,^13^,^36^ In contrast, MDD was not found to serve as a risk factor for the development of GAD, which is at odds with at least one study that examined the relations among pure and co-morbid GAD and MDD.^12^

Limitations of the present study should be considered when interpreting the findings. Although the present study is one of the first to examine the stability and longitudinal associations of both anxiety and depression during the postpartum period, the investigation was limited to GAD and MDD. As other manifestations of depression and anxiety (e.g., panic disorder and obsessive compulsive disorder^40^) exist during the postpartum period, it is important to investigate these syndromes as well. Also, rates of GAD and MDD in the present sample were greater than that of the typical maternal population, and the no diagnosis group at 3M-P was selected such that participants had no history of psychiatric diagnosis, both of which alter the effect sizes of the observed relations. Future work should extend these findings by examining them in a more representative sample of postpartum mothers. Another limitation is that variables that were not controlled for, such as medication use or psychotherapy, could have influenced diagnostic status and symptom levels. It is recommended that future studies account for the influence of such variables. Lastly, the EPDS and GAD-Q questionnaires were administered 2–3 weeks before the 3M-P SCID interview.

Despite these limitations, the present findings are the first to investigate the course and longitudinal associations between GAD and MDD during the postpartum period. The temporal specificity of GAD and MDD during this period, in addition to the significant differences in stability and longitudinal associations, provide additional evidence for the argument that GAD and MDD are distinct disorders. Further, because GAD was found to be a potential risk factor for subsequent MDD during this period, interventions could be designed to both treat maternal GAD and prevent future MDD. The present findings also revealed that co-morbid GAD + MDD is the most stable and the least likely to be associated with subsequent periods free from diagnosis. Given these findings and evidence demonstrating the negative effects of maternal depression^1^,^14^–^19^ and anxiety^37^ on childhood development, and the fact that the risk of adverse child outcomes are increased when postnatal depression persists,^17^ it is vital to screen for and treat new mothers who are suffering from both GAD and MDD.

## Footnotes

1 Figure 1 odds ratios are based on actual cell frequencies. However, certain analyses had zero count cells resulting in untrustworthy standard errors. For these instances (indicated by odds ratios of zero in Figure 1), a value of 1 was added to each cell in the contingency table to provide more stable standard errors for significance testing.

2 Continuous subscales were created and used as indicators of the latent constructs rather than categorical item-level indicators to decrease the number of estimated parameters. Two subscales were created to provide each latent construct with three indicators because having three indicators improved model convergence rates. Two items from the EPDS were used as indicators of GAD symptoms as exploratory (EFAs) and confirmatory factor analyses (CFAs) revealed that these items loaded on an anxiety factor along with the GAD-Q items and this factor was significantly associated with GAD CSRs. These items involved being “anxious or worried” and feeling “scared or panicky” for no good reason. Six of the remaining eight EPDS items were chosen as indicators of MDD symptoms as EFA and CFA revealed that they loaded on a single factor and were significantly associated with MDD CSRs. Additional details are available upon request.

3 These partially metric invariant models were used for subsequent analyses because applying across time equality constraints for full metric invariance resulted in a significant decrement in model fit (as noted above). However, applying all equality constraints for subsequent analyses did not appreciably change the results: all models still fit well, the same pattern of significance was seen for model comparisons, and there was little change in model parameter estimates.
